# Supplementary material for: Activation of CAMK2 by pseudokinase PEAK1 represents a targetable pathway in triple negative breast cancer
Source: Nat Commun. 2025 Feb 22;16:1871. doi: 10.1038/s41467-025-57046-8 (PMC11845518; doi:10.1038/s41467-025-57046-8)

Supplementary Information for 'Activation of CAMK2 by pseudokinase PEAK1 represents a targetable pathway in triple negative breast cancer' by Yang *et al*

A

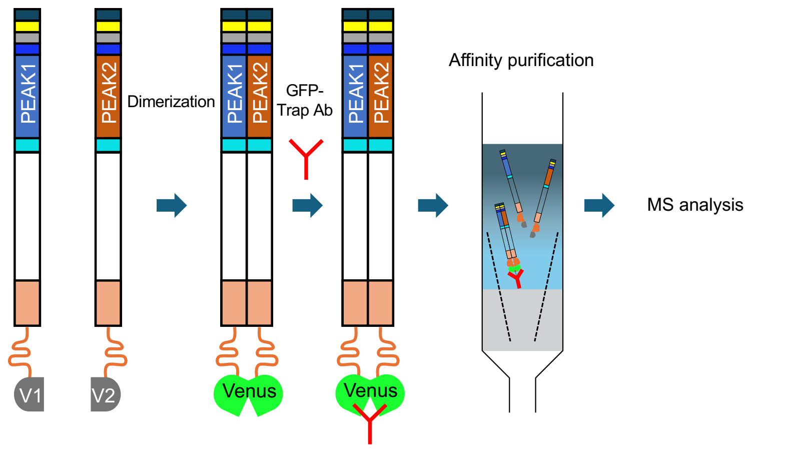

B

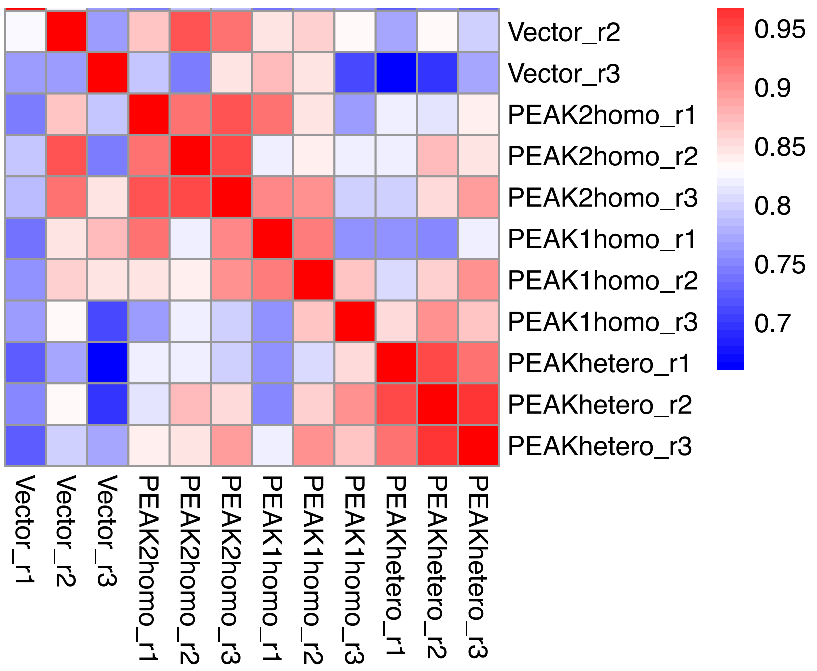

Supp Figure 1. Characterization of PEAK complex interactomes via BiCAP-MS/MS. A. Schematic strategy of screen. This is indicated for the PEAK1/PEAK2 heterodimer. PEAK1 and PEAK2 were fused to N-terminal (V1) and C-terminal (V2) fragments of the Venus protein, respectively. When PEAK1 and PEAK2 associate, V1 and V2 bind to each other and reconstitute Venus that can be recognized by the GFP-targeting nanobody (GFP-Trap). The heterodimer and its interactome can then be immunoaffinity purified for MS analysis. In the screen, this workflow was applied to the PEAK1 and PEAK2 homodimers as well as the

heterodimer. **B. Sample correlation heatmap for the DIA MS analyses.** Data shown are for n=3 independent experiments, r1-r3. See Data Availability section for access to source data.

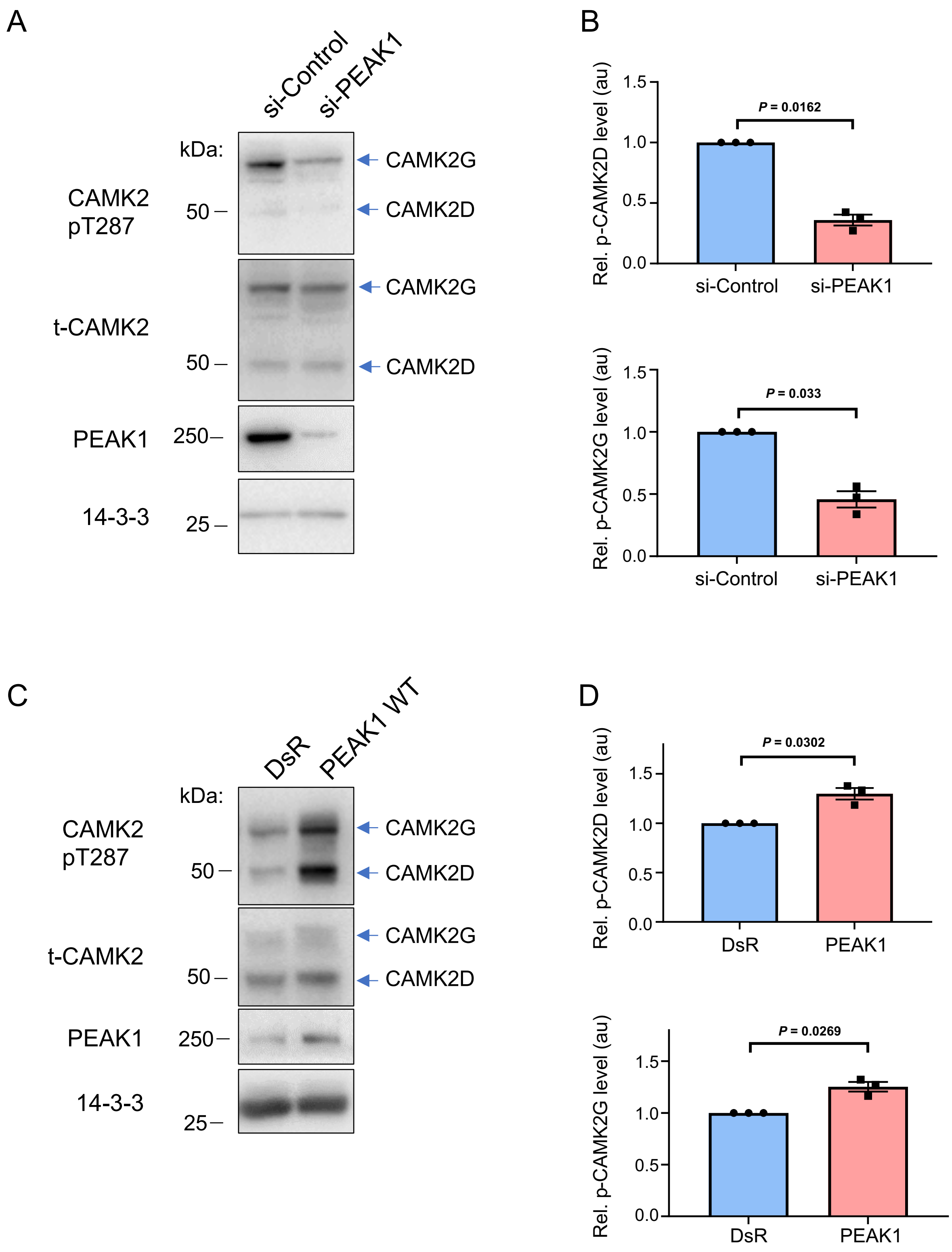

Supp Figure 2. Manipulation of PEAK1 expression modulates CAMK2 activity in MDA-MB-468 cells. PEAK1 was knocked down using siRNA (A-B) or overexpressed via transient transfection (C-D) in MDA-MB-468 cells and autonomous activation of CAMK2 determined by Western blotting as indicated. Histograms indicate CAMK2 T287 phosphorylation normalized for total CAMK2 expression, with 'au' indicating arbitrary units. Data are presented as mean values +/- standard error of the mean (SEM) from n=3 independent experiments and analysed by ratio paired two-tailed t-test. Source data are provided as a Source Data file. Uncropped scans of blots are provided at the end of Supplementary Information.

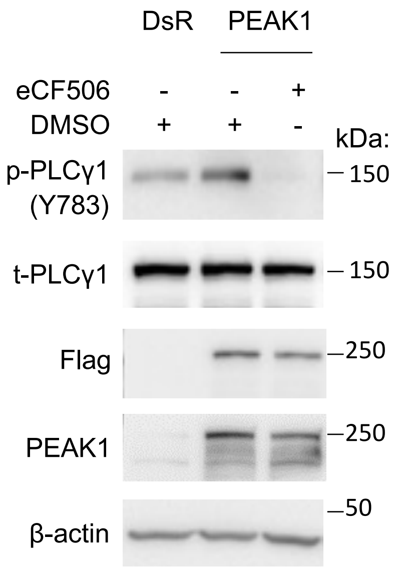

Supp Figure 3. Effect of SFK inhibitor eCF506 on PLCγ1 Y783 phosphorylation. MDA-MB-231_EcoR cells stably transduced with DsR empty vector or the corresponding PEAK1 construct were treated with DMSO or the SFK inhibitor eCF506 (250 nM) for 1 h. Cell lysates were then analysed by Western blotting as indicated. Data are representative of n=2 independent experiments. Uncropped scans of blots are provided at the end of Supplementary Information.

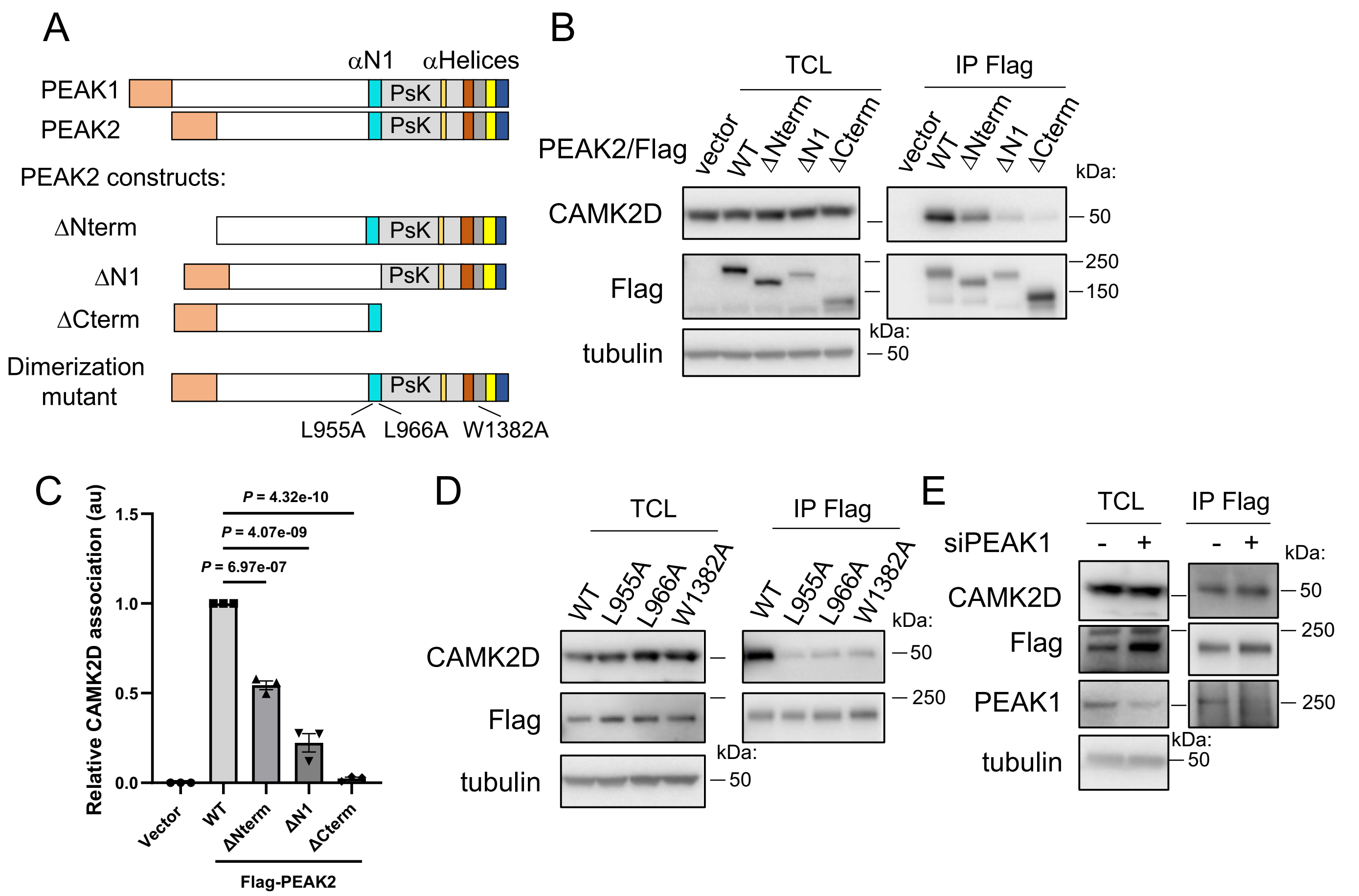

Supp Figure 4. Determination of the structural requirements for CAMK2 interaction with PEAK2. A. Schematic structure of PEAK2 and various deletion and point mutants. **B. Identification of PEAK2 regions required for association with CAMK2D.** Flag-tagged versions of the indicated PEAK2 proteins were transfected into HEK293T cells. Total cell lysates (TCL) and Flag immunoprecipitates (IPs) were subjected to Western blotting. Data are representative of n=3 independent experiments. **C. Quantification of CAMK2D association.** CAMK2D binding to PEAK2 was quantified and normalized for the PEAK2 level. Data are expressed relative to WT which was arbitrarily set at 1, with 'au' indicating arbitrary units. Data represent the mean ± SEM of n=3 independent experiments and were analysed by one-way ANOVA with Dunnett's multiple comparisons test. **D. Role of PEAK2 dimerization in regulating association with CAMK2D.** Flag-tagged versions of the indicated PEAK2 proteins were expressed in HEK293T cells. TCL and Flag IPs were subjected to Western blotting as indicated. **E. The association of PEAK2 with CAMK2D is PEAK1-independent.** HEK293T cells were transfected with a plasmid encoding Flag-tagged PEAK2 in the presence or absence of siRNA-mediated PEAK1 knockdown. TCL and Flag IPs were subjected to Western blotting as indicated. Data in D-E are representative of n=2 independent experiments. Source data are provided as a Source Data file. Uncropped scans of blots are provided at the end of Supplementary Information.

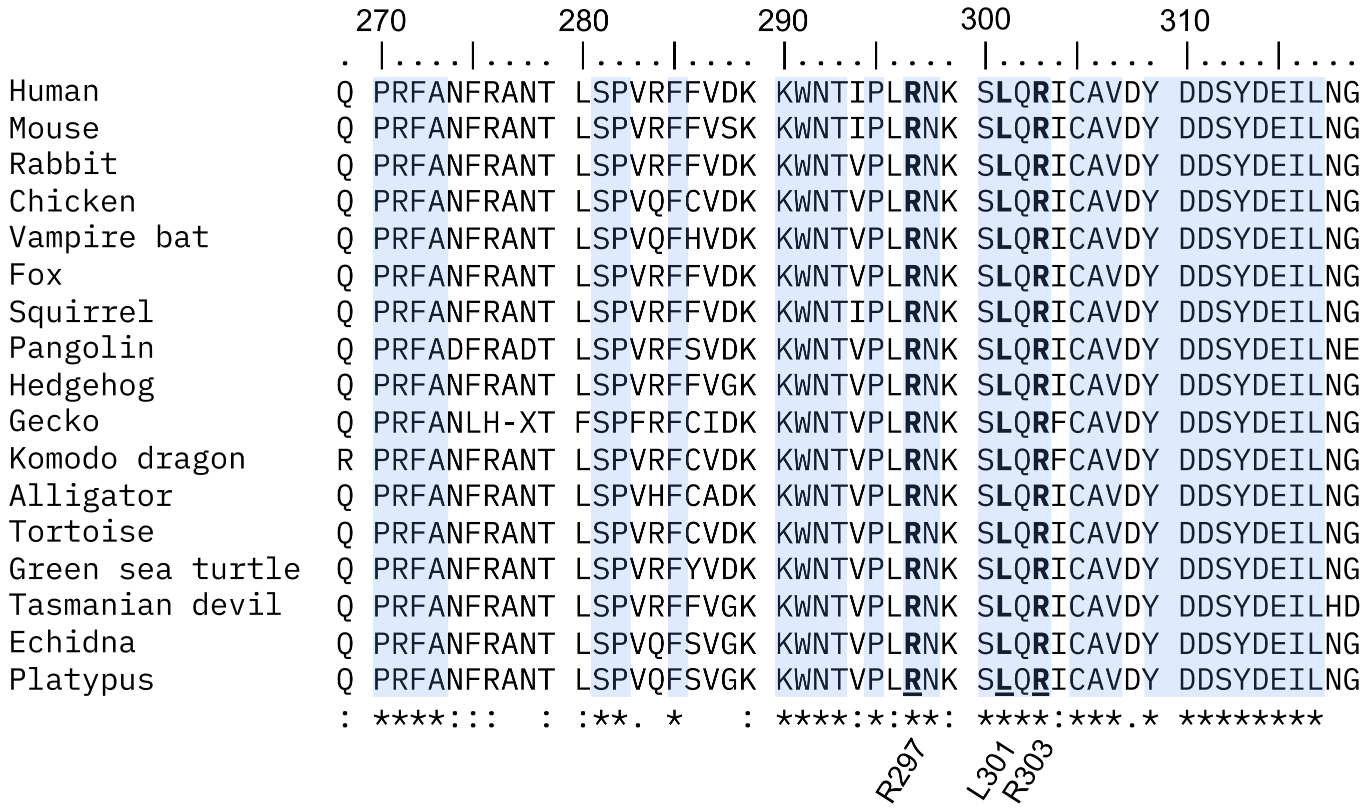

Supp Figure 5. Sequence alignment of the PEAK1 CIM motif across diverse species. The critical residues for CAMK2 interaction, R297, L301 and R303 are conserved across species and highlighted.

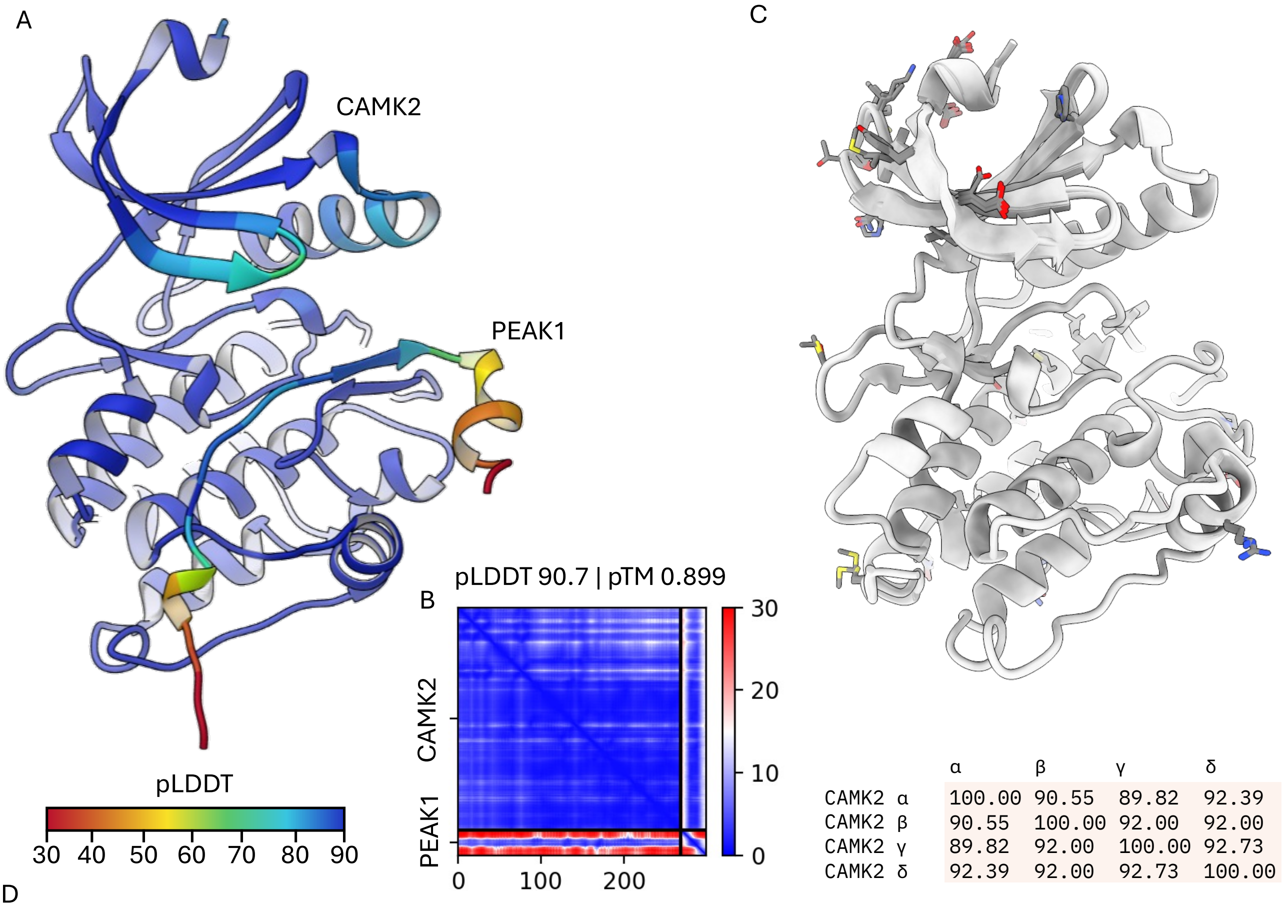

	α	β	γ	δ
CAMK2 α	100.00	90.55	89.82	92.39
CAMK2 β	90.55	100.00	92.00	92.00
CAMK2 γ	89.82	92.00	100.00	92.73
CAMK2 δ	92.39	92.00	92.73	100.00

D

```
CAMK2 α   TRFTEEYQLFEELGKGAFSVVRRCVKVLAGQEYAAKIINTKKLSARDHQKLEREARICRLLKHPNIVRLHDSISEEGHHYLIFDLVTGGELFEDI 101
CAMK2 β   -RFTDEYQLYEDIGKGAFSVVRRCVKLCTGHEYAAKIINTKKLSARDHQKLEREARICRLLKHSNIVRLHDSISEEGFHYLVFDLVTGGELFEDI 102
CAMK2 γ   -RFTDDYQLFEELGKGAFSVVRRCVKKTSTQEYAAKIINTKKLSARDHQKLEREARICRLLKHPNIVRLHDSISEEGFHYLVFDLVTGGELFEDI 102
CAMK2 δ   TRFTDEYQLFEELGKGAFSVVRRCMKIPTGQEYAAKIINTKKLSARDHQKLEREARICRLLKHPNIVRLHDSISEEGFHYLVFDLVTGGELFEDI 102
           ***::***:*::***********:*  : :******************************** *************.***:************

CAMK2 α   VAREYYSEADASHCIQQILEAVLHCHQMGVVHRDLKPENLLLASKLKGAAVKLADFGLAIEVEGEQQAWFGFAGTPGYLSPEVLRKDPYGKPVDL 196
CAMK2 β   VAREYYSEADASHCIQQILEAVLHCHQMGVVHRDLKPENLLLASKCKGAAVKLADFGLAIEVQGDQQAWFGFAGTPGYLSPEVLRKEAYGKPVDI 197
CAMK2 γ   VAREYYSEADASHCIHQILESVNHIHQHDIVHRDLKPENLLLASKCKGAAVKLADFGLAIEVQGEQQAWFGFAGTPGYLSPEVLRKDPYGKPVDI 197
CAMK2 δ   VAREYYSEADASHCIQQILESVNHCHLNGIVHRDLKPENLLLASKSKGAAVKLADFGLAIEVQGDQQAWFGFAGTPGYLSPEVLRKDPYGKPVDM 197
          ***************:****:* * *  .:*************** ****************:*:**********************: ******:

CAMK2 α   WACGVILYILLVGYPPFWDEDQHRLYQQIKAGAYDFPSPEWDTVTPEAKDLINKMLTINPSKRITAAEALKHPWISHRSTVASCMHR      283
CAMK2 β   WACGVILYILLVGYPPFWDEDQHKLYQQIKAGAYDFPSPEWDTVTPEAKNLINQMLTINPAKRITAHEALKHPWVCQRSTVASMMH-      283
CAMK2 γ   WACGVILYILLVGYPPFWDEDQHKLYQQIKAGAYDFPSPEWDTVTPEAKNLINQMLTINPAKRITADQALKHPWVCQRSTVASMMH-      283
CAMK2 δ   WACGVILYILLVGYPPFWDEDQHRLYQQIKAGAYDFPSPEWDTVTPEAKDLINKMLTINPAKRITASEALKHPWICQRSTVASMMH-      283
          ***********************:*************************:***:******:*****  :******:.:****** **
```

Supp Figure 6. Comparative modelling analysis by AlphaFold (to accompany Figure 4C-F). A. Computationally predicted PEAK1(291-320)/CAMK2 model by AlphaFold (v3) coloured according to pLDDT score. Overall pLDDT and pTM scores of 90.7 and 0.899 indicate high estimated confidence measured by per-residue local distance difference (LDDT) and residue-residue alignment error estimate respectively. **B. Predicted alignment error plot illustrating low overall estimated error between PEAK1(291-320) and CaMK2 (top right quadrant). C. Superimposition of CAMK2 isoforms.** Residues with less than 100% identity are shown as sticks. All other residues (identity 100%) are hidden for clarity. The residues that define the PEAK1(291-320)/CAMK2 interaction are conserved across all isoforms (α, β, γ, and δ). The identity matrix is shown below, with all isoforms having ~ 90% identity. **D**. Multiple sequence alignment of the domain region shown in (A) highlighting very high conservation between CAMK2 isoforms.

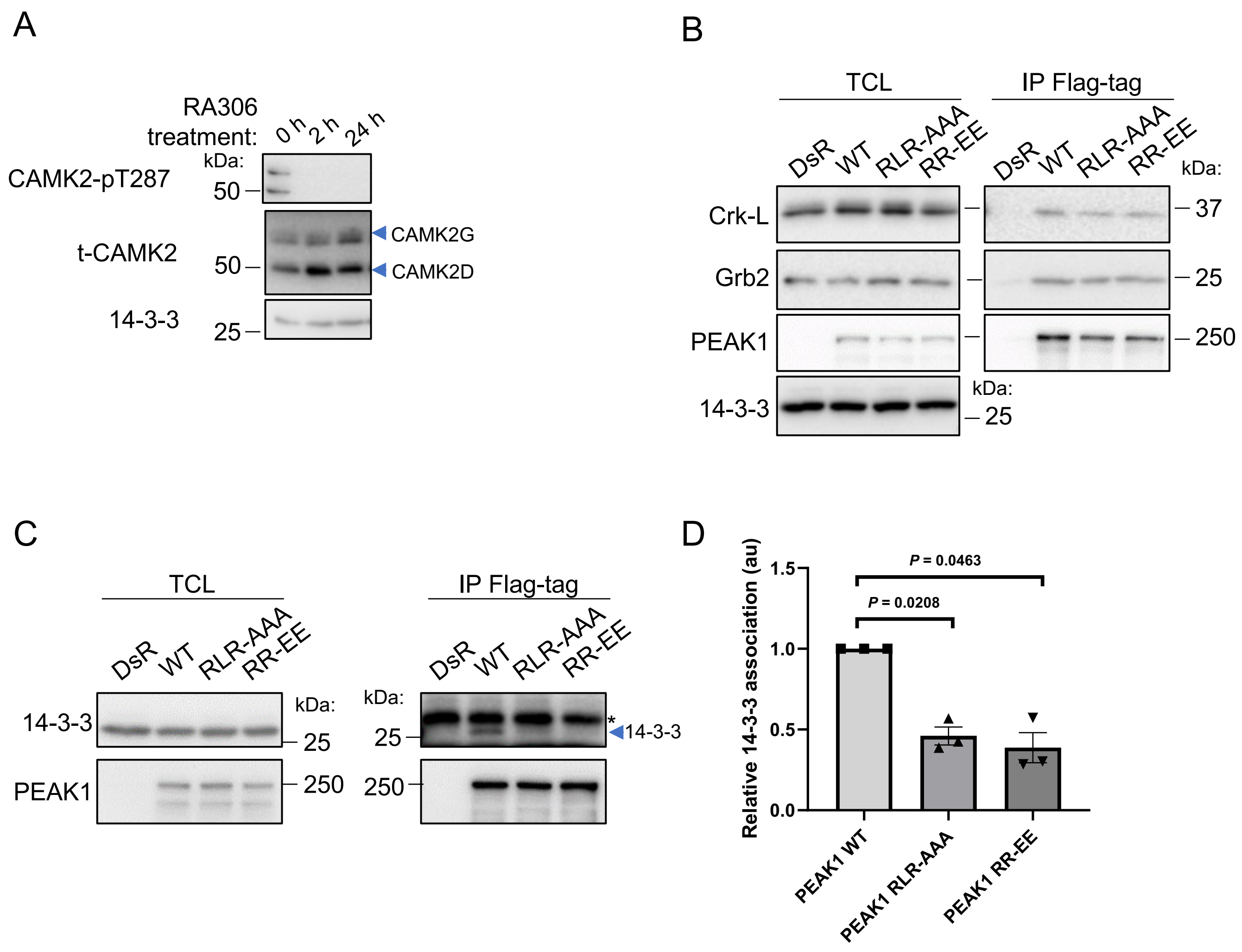

Supp Figure 7. Role of CAMK2 in regulating PEAK1 phosphorylation and the PEAK1 interactome. A. Validation of CAMK2 inhibition using RA306. MDA-MB-231 cells were treated with 1 µM RA306 for the specified times and then cell lysates were Western blotted as indicated. Data are representative of n=3 independent experiments. **B-D. Impact of CIM mutations on the PEAK1 interactome.** IPs of WT and mutant PEAK1 proteins from HEK293T cells were analysed by immunoblotting with the indicated antibodies. Results are representative of n=3 independent experiments. The asterisk in C indicates a non-specific band. Histogram (D) indicates the association of 14-3-3 relative to WT PEAK1 (mean ± SEM, n = 3), with 'au' indicating arbitrary units and significance by ratio paired two-tailed t-test. Source data are provided as a Source Data file. Uncropped scans of blots are provided at the end of Supplementary Information.

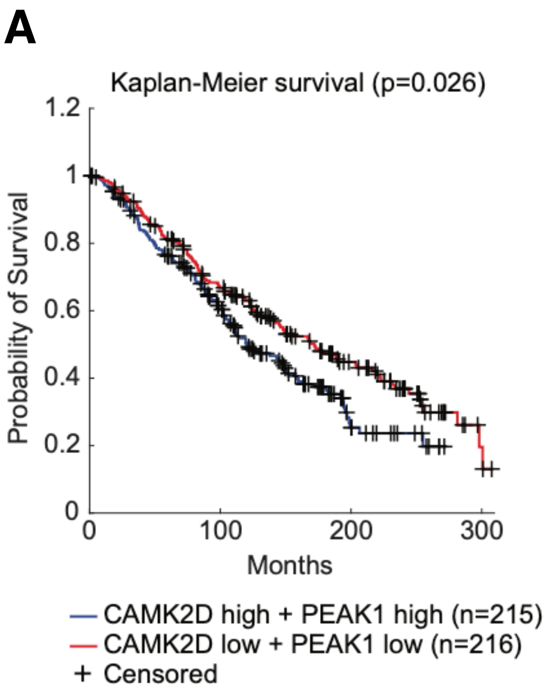

B

Comparison

CAMK2D/PEAK1	CAMK2D/PEAK1	P-value
high/high	low/low	**0.026**
low/high	low/low	**0.8**
high/high	high/low	**0.051**
high/low	low/low	0.71
high/high	low/high	**0.026**

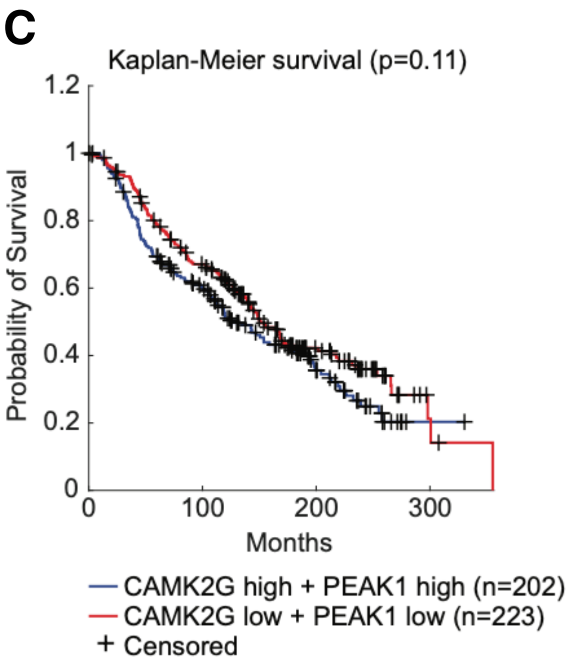

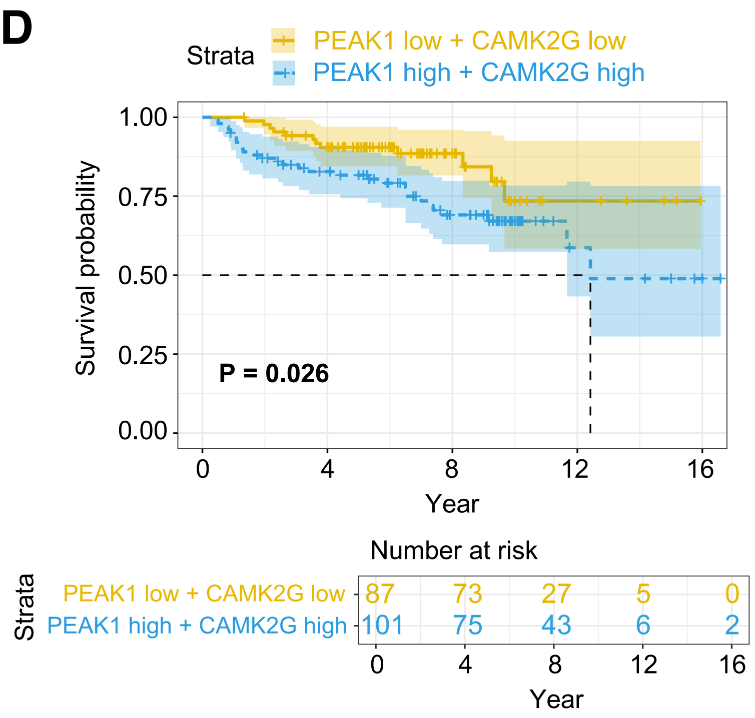

Supp Figure 8. Association of PEAK1 and CAMK2 expression with breast cancer patient survival. Gene expression, mutation profile and associated overall survival data from 2509 breast cancer patients were downloaded from the cBioPortal for Cancer Genomics portal (https://www.cbioportal.org/). Breast cancer patients were categorised into groups exhibiting different PEAK1/CAMK2 combined expression patterns. **A. Survival analyses comparing overall survival between PEAK1/CAMK2D both high and PEAK1/CAMK2D both low.** This was undertaken using a Log-rank test. **B. Summary table comparing overall survival differences between indicated PEAK1/CAMK2D combined expression patterns. C. Survival analyses comparing overall survival between PEAK1/CAMK2G both high and PEAK1/CAMK2G both low patient groups.** The Log-rank test statistics and survival curves were generated

using Kaplan-Meier estimate and implemented using the Logrank package in MATLAB 2023a (with $p < 0.05$ considered significant). **D. Distant metastasis-free survival (DMFS) for the PEAK1/CAMK2G both high and PEAK1/CAMK2G both low patient groups.** DMFS was compared between the two subgroups using a log-rank test (with $p < 0.05$ considered significant). For cohort details, please refer to Methods.

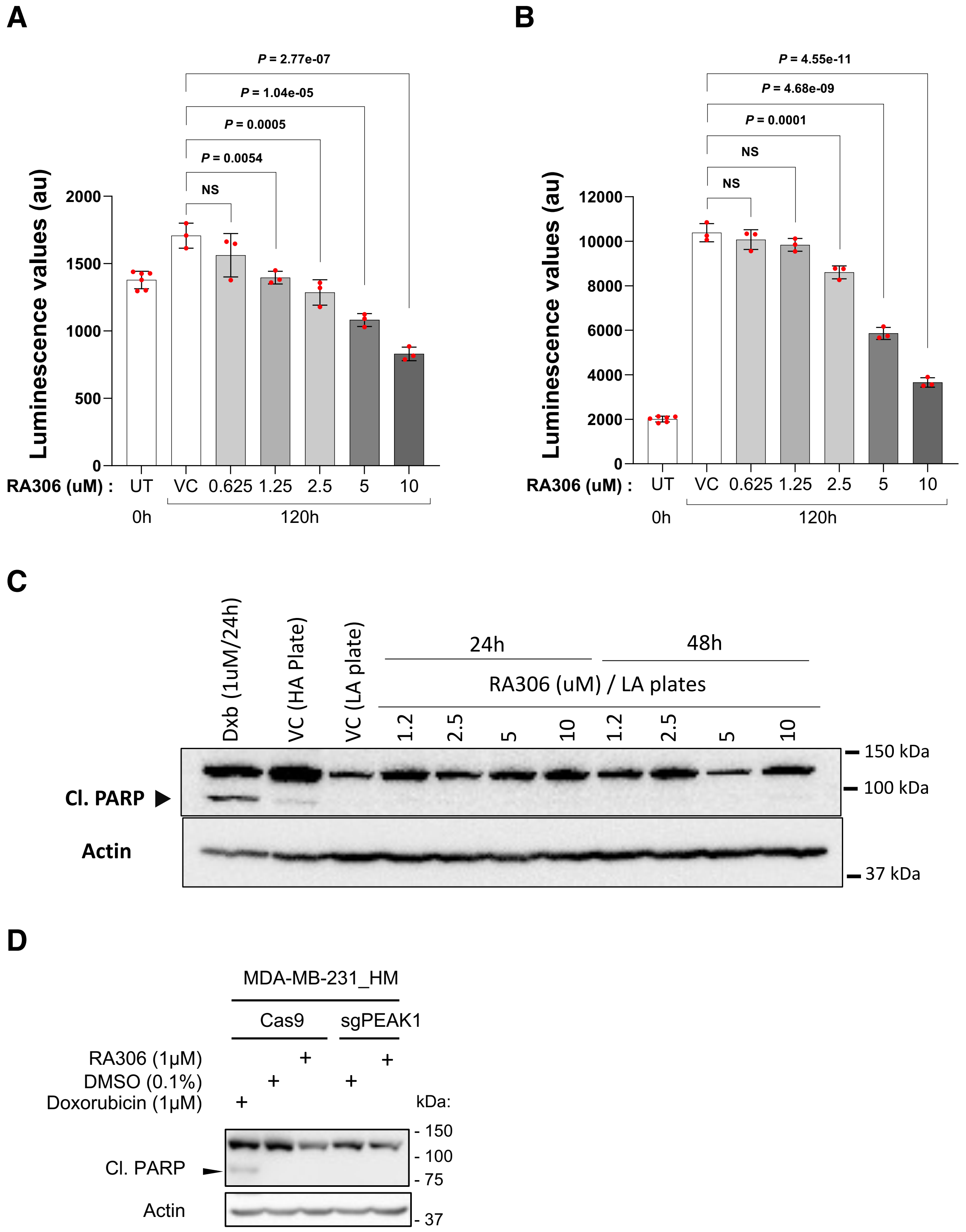

Supp Figure 9. Effect of the CAMK2 inhibitor RA306 on cell proliferation and apoptosis. A-B, Effect on cell proliferation under low (A) and high (B) attachment conditions. Cell viability assays were undertaken at the indicated time points, with data points indicating mean +/- standard deviation for n=3 independent experiments. UT, untreated; VC, vehicle control (DMSO); au, arbitrary units. ns = not significant at $p<0.05$. Data were analysed by one-way ANOVA with Dunnett's Multiple Comparison test. **C. Effect of**

RA306 on apoptosis under low attachment conditions. MDA-MB-231 HM cell lysates were prepared at the indicated time after plating into low attachment plates and Western blotted as indicated. Doxorubicin (Dxb) treatment of cells on high attachment plates was used as a positive control for induction of cleaved (Cl) PARP. VC, vehicle control (DMSO) for 48h on high attachment (HA) and low attachment (LA) plates. Data are representative of n=2 independent experiments. **D. Effect of RA306 and PEAK1 gene knockout on apoptosis.** Control (Cas9) or PEAK1 gene knock-out (sgPEAK1) cells on HA plates were treated with vehicle control (DMSO) or RA306 for 24 h. Cell lysates were then Western blotted as indicated. Doxorubicin treatment was used as a positive control for induction of cleaved (Cl) PARP. Data are representative of n=2 independent experiments. Source data are provided as a Source Data file. Uncropped scans of blots are provided at the end of Supplementary Information.

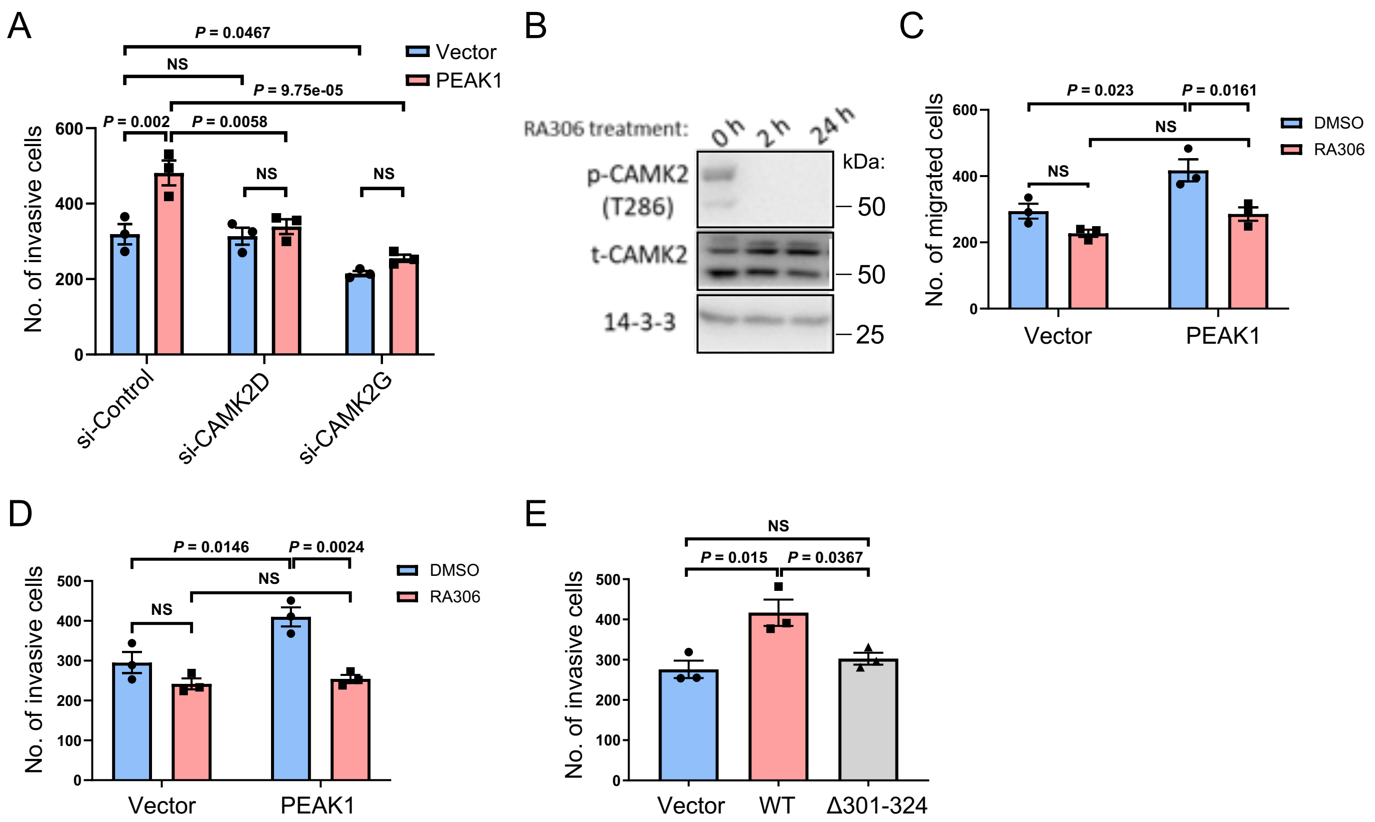

Supp Figure 10. Role of CAMK2 in PEAK1-regulated biological endpoints in TNBC models. A. Role of CAMK2D/G in PEAK1-promoted MDA-MB-231 cell invasion. MDA-MB-231 cells were transfected with a PEAK1 expression plasmid in the presence or absence of siRNA-mediated CAMK2D or CAMK2G knockdown. Cell lysates were validated by Western blotting in Figure 7A. Cells were subjected to a transwell invasion assay. **B-D. Pharmacological inhibition of CAMK2 using RA306 blocks PEAK1-promoted TNBC cell migration and invasion.** MDA-MB-468 cells were treated with RA306 for different times and cell lysates were then Western blotted as indicated (B). Data are representative of n=2 independent experiments. MDA-MB-468 cells were transfected with a PEAK1 plasmid and subject to transwell migration assays in the presence or absence of RA306 (C). MDA-MB-231 cells were transfected with a PEAK1 plasmid and subjected to transwell invasion assays in the presence or absence of RA306 (D). **E. Role of CAMK2 activation.** Plasmids expressing WT PEAK1 and the Δ301-324 mutant that cannot activate CAMK2 were transiently transfected into MDA-MB-231 cells, and cells were subjected to a transwell invasion assay. Data are presented as mean +/- SEM from n=3 independent experiments. NS indicates $p > 0.05$. Data were analysed by two-way ANOVA with Tukey's multiple comparisons test (A, C, D) or one-way ANOVA with Tukey's multiple comparisons test (E). Source data are provided as a Source Data file. Uncropped scans of blots are provided at the end of Supplementary Information.

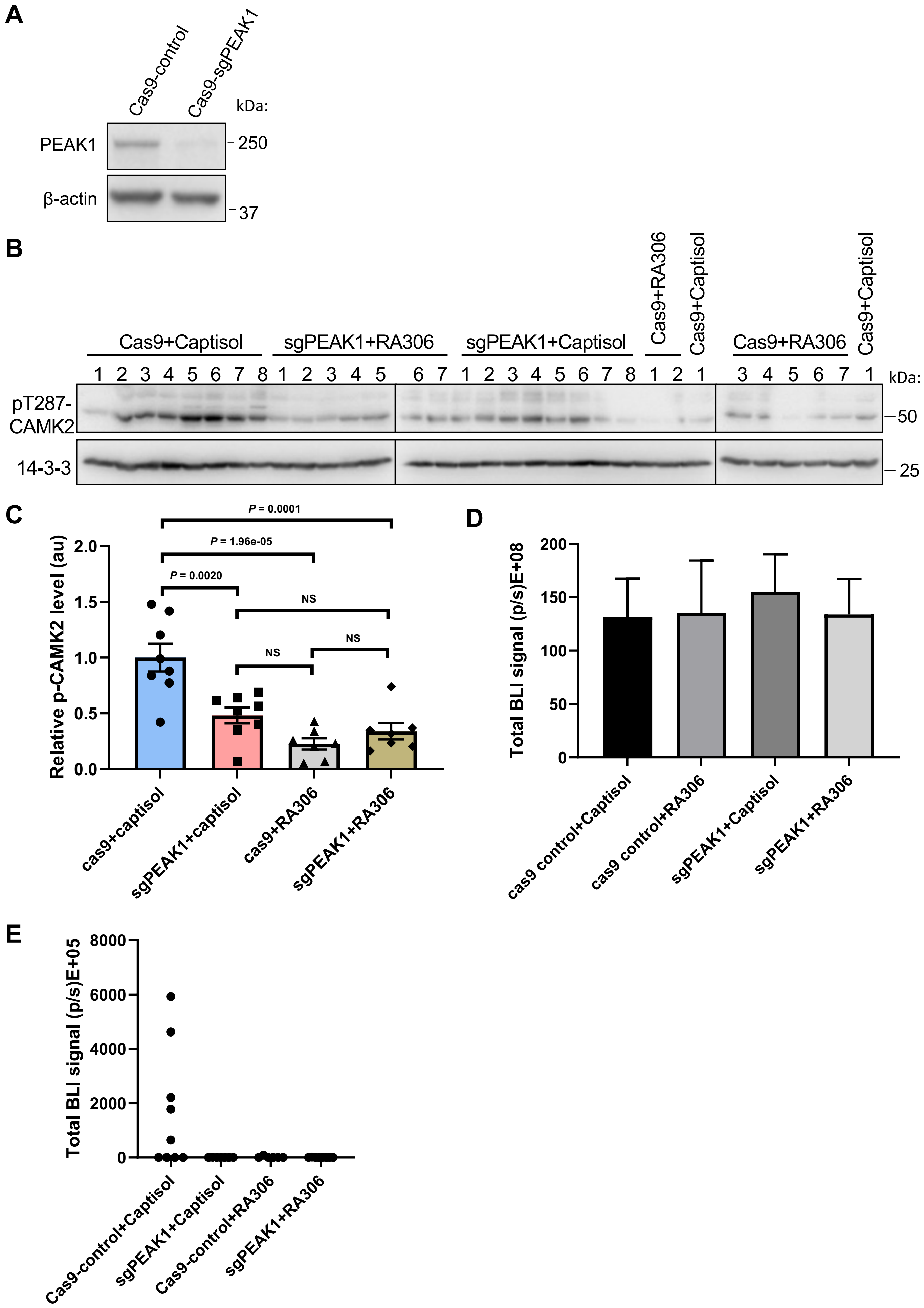
A
Cas9-control
Cas9-sgPEAK1
kDa:
PEAK1
250
β-actin
37
B
Cas9+Captisol
sgPEAK1+RA306
sgPEAK1+Captisol
Cas9+RA306
Cas9+Captisol
Cas9+RA306
Cas9+Captisol
1 2 3 4 5 6 7 8 1 2 3 4 5 6 7 1 2 3 4 5 6 7 8 1 2 1 3 4 5 6 7 1
kDa:
pT287-CAMK2
50
14-3-3
25
C
P = 0.0001
P = 1.96e-05
P = 0.0020
NS
NS
NS
Relative p-CAMK2 level (au)
2.0
1.5
1.0
0.5
0.0
cas9+captisol
sgPEAK1+captisol
cas9+RA306
sgPEAK1+RA306
D
Total BLI signal (p/s)E+08
200
150
100
50
0
cas9 control+Captisol
cas9 control+RA306
sgPEAK1+Captisol
sgPEAK1+RA306
E
Total BLI signal (p/s)E+05
8000
6000
4000
2000
0
Cas9-control+Captisol
sgPEAK1+Captisol
Cas9-control+RA306
sgPEAK1+RA306

Supp Figure 11. Genetic or pharmacological targeting of the PEAK1/CAMK2 axis reduces TNBC development *in vivo*. A. Validation of CRISPR-mediated PEAK1 knockout in MDA-MB-231_HM cells. Cells were Western blotted as indicated. Data are representative of n=2 independent experiments. **B-C. CAMK2 activation in different xenograft treatment groups.** Tumour tissue from each group was harvested at 2 h after last RA306 treatment, homogenized and subjected to Western blotting (B) and quantification for combined CAMK2D and CAMK2G activation (C) as indicated. In C, data are expressed relative to Cas9-control/Captisol which was arbitrarily set at 1. Mouse tumour sample numbers were: Cas9 control + captisol, 8; sgPEAK1 + captisol, 8; Cas9 control + RA306, 7; sgPEAK1 + RA306, 7. Data are presented as mean +/- SEM. NS indicates $p > 0.05$. Data were analysed by two-way ANOVA with Tukey's multiple comparisons test. **D. Primary tumour sizes prior to resection for metastasis experiment.** This accompanies Figure 7F. Data points are mean +/- SEM. **E.** Luciferase intensities of tumour metastasis to lung for each treatment group. This accompanies Figure 7F. Source data are provided as a Source Data file. Uncropped scans of blots are provided at the end of Supplementary Information.

Uncropped blots for figures provided in Supplementary Information are provided in the following section.

Supp Fig 2A

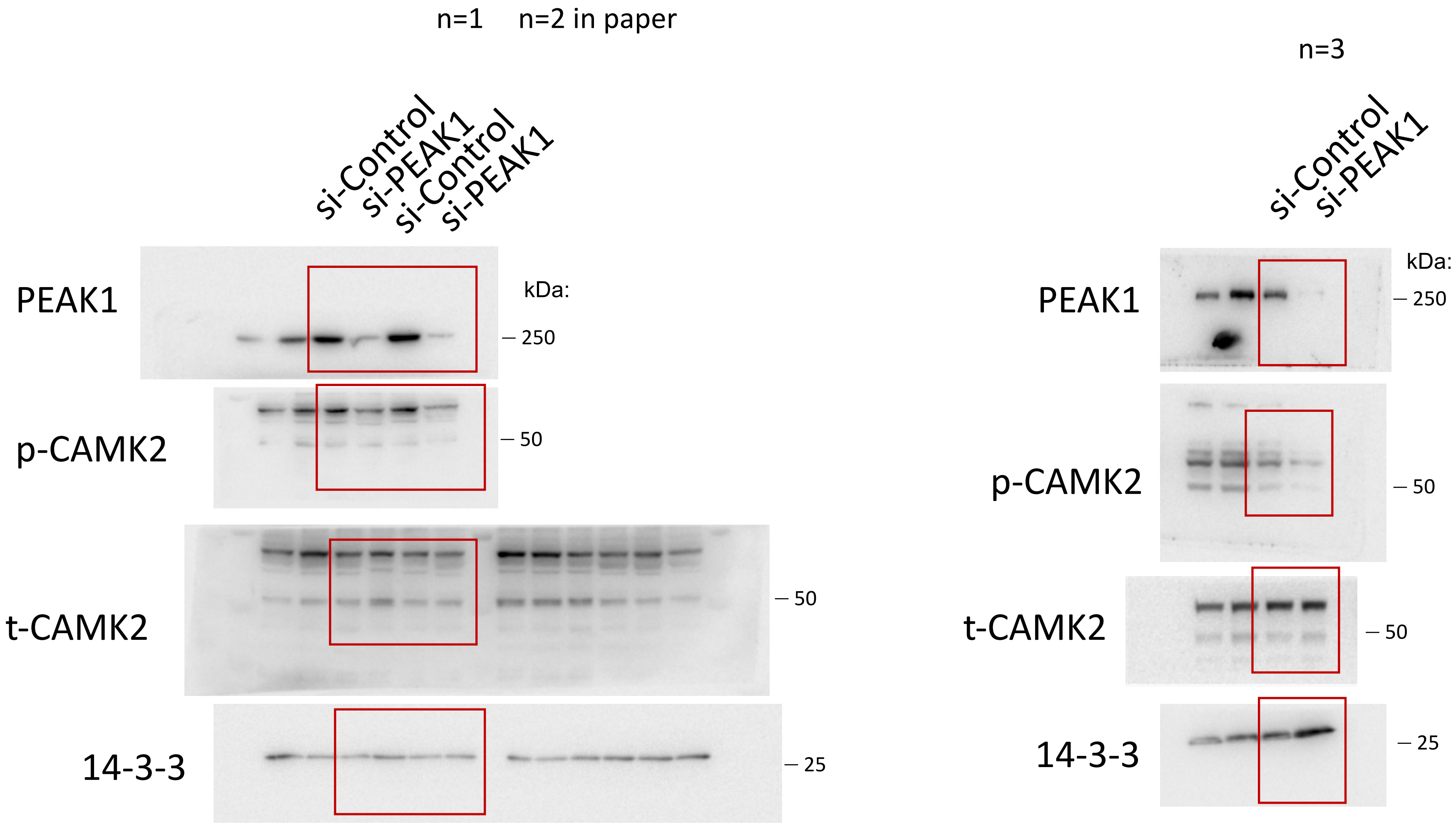
n=1
n=2 in paper
si-Control
si-PEAK1
si-Control
si-PEAK1
PEAK1
kDa:
250
p-CAMK2
50
t-CAMK2
50
14-3-3
25
n=3
si-Control
si-PEAK1
PEAK1
kDa:
250
p-CAMK2
50
t-CAMK2
50
14-3-3
25

Supp Fig 2C

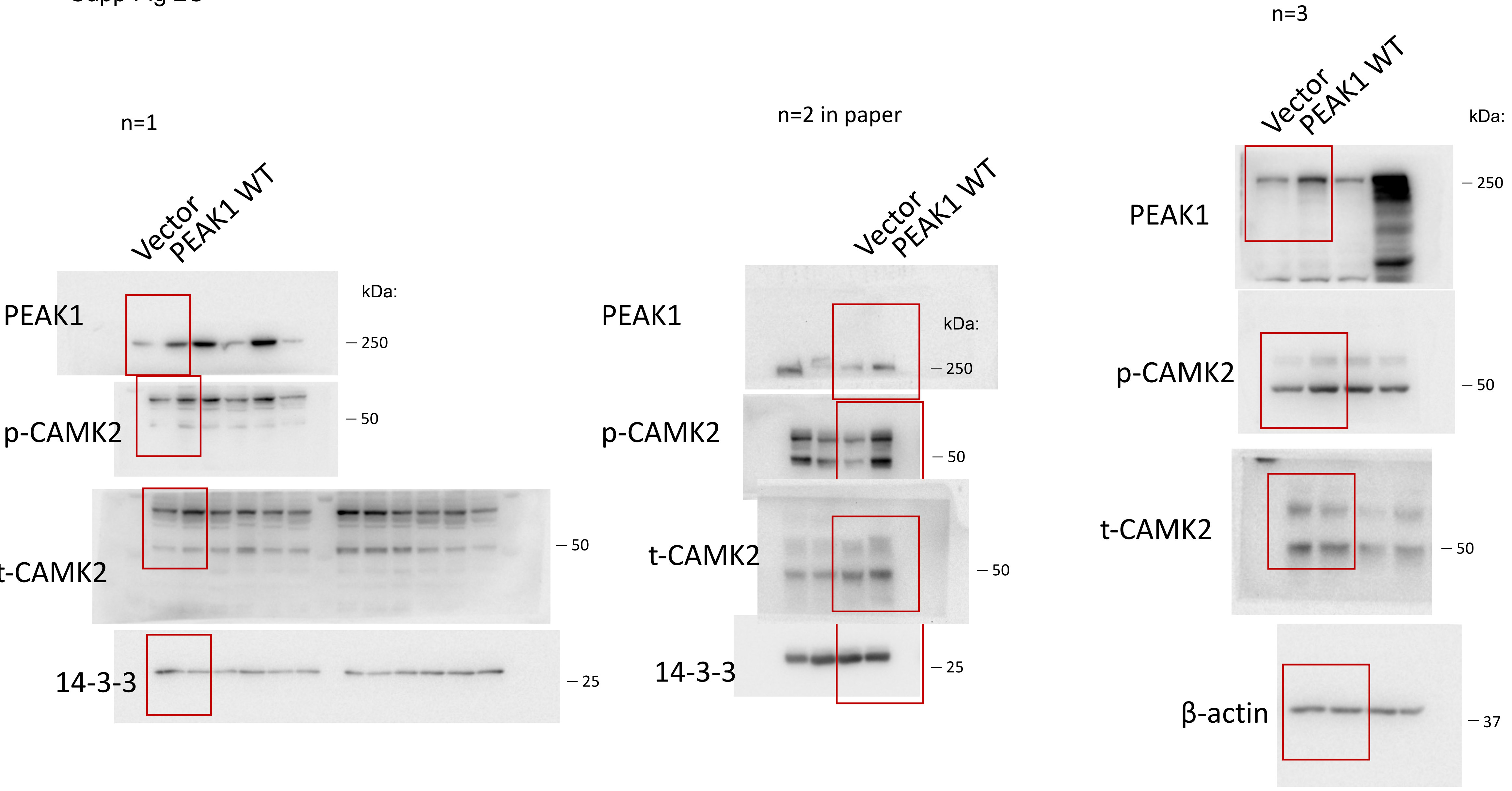
n=1
Vector
PEAK1 WT
PEAK1
kDa:
250
p-CAMK2
50
t-CAMK2
50
14-3-3
25
n=2 in paper
Vector
PEAK1 WT
PEAK1
kDa:
250
p-CAMK2
50
t-CAMK2
50
14-3-3
25
n=3
Vector
PEAK1 WT
kDa:
PEAK1
250
p-CAMK2
50
t-CAMK2
50
β-actin
37

Supp Fig 3

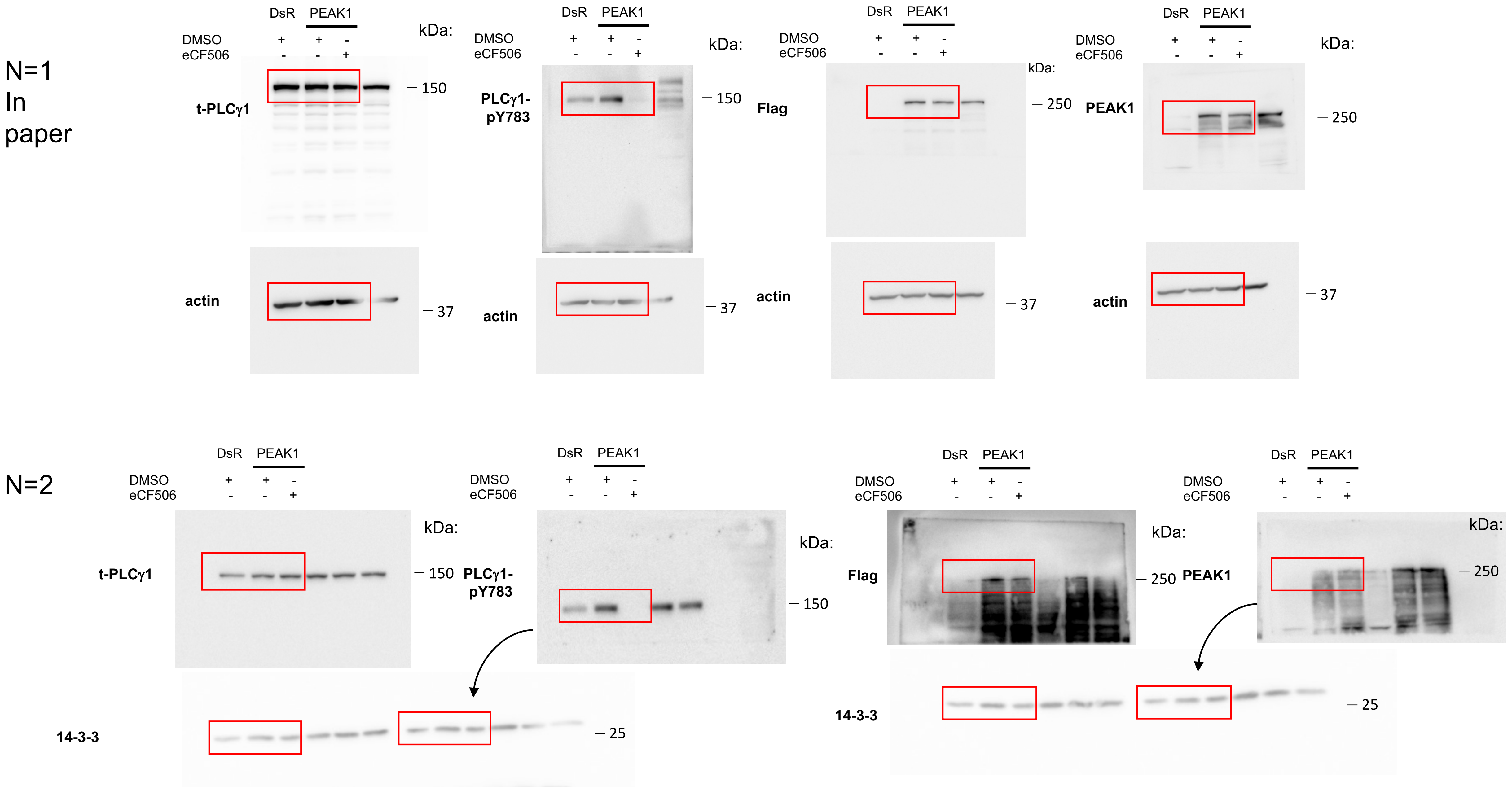
N=1
In paper
DsR
PEAK1
DMSO
eCF506
kDa:
t-PLCγ1
150
actin
37
PLCγ1-pY783
Flag
250
PEAK1
N=2
14-3-3
25

Supp Fig. 4b

n=1, Shown in paper

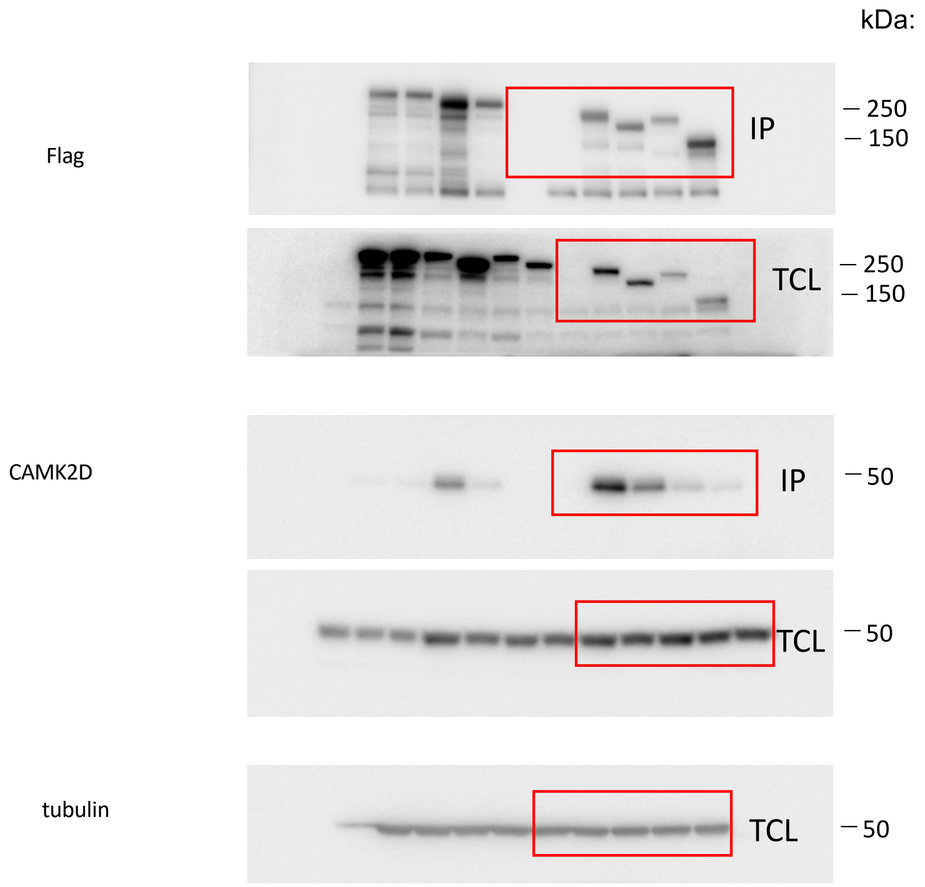

Supp Fig. 4b

n=2

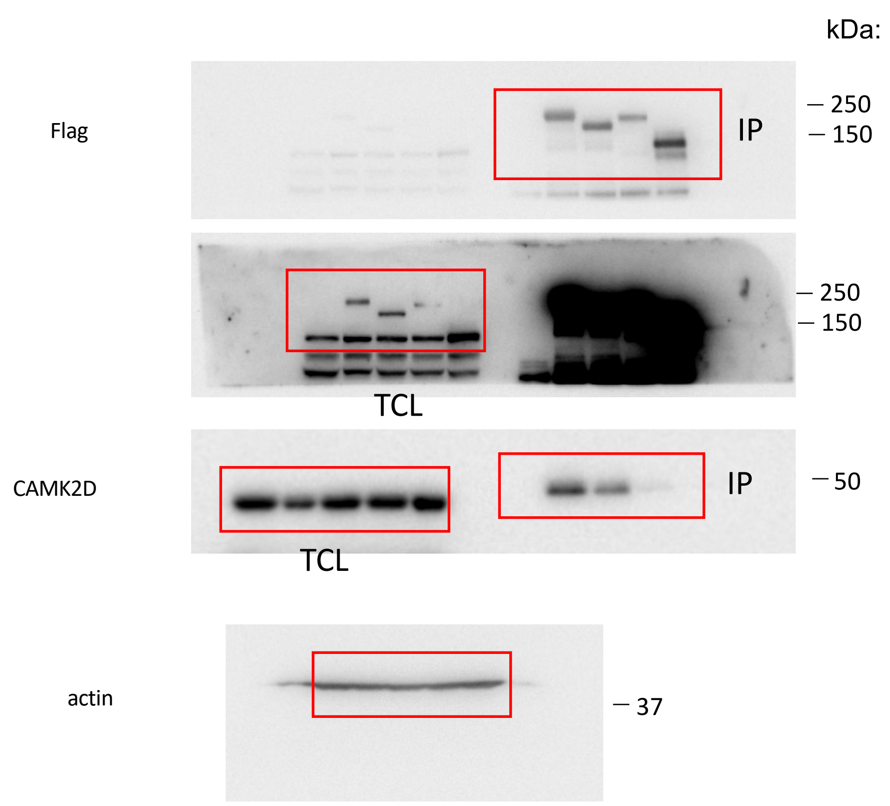

Supp Fig. 4b

n=3

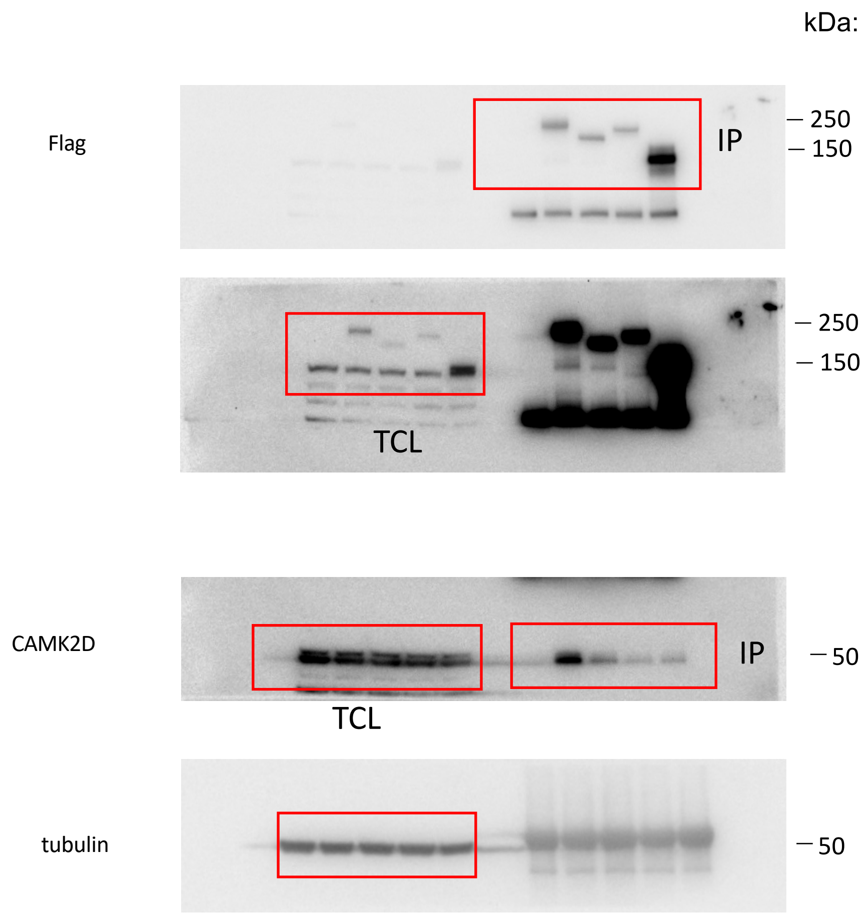

Supp Fig. 4D

n=1, Shown in paper

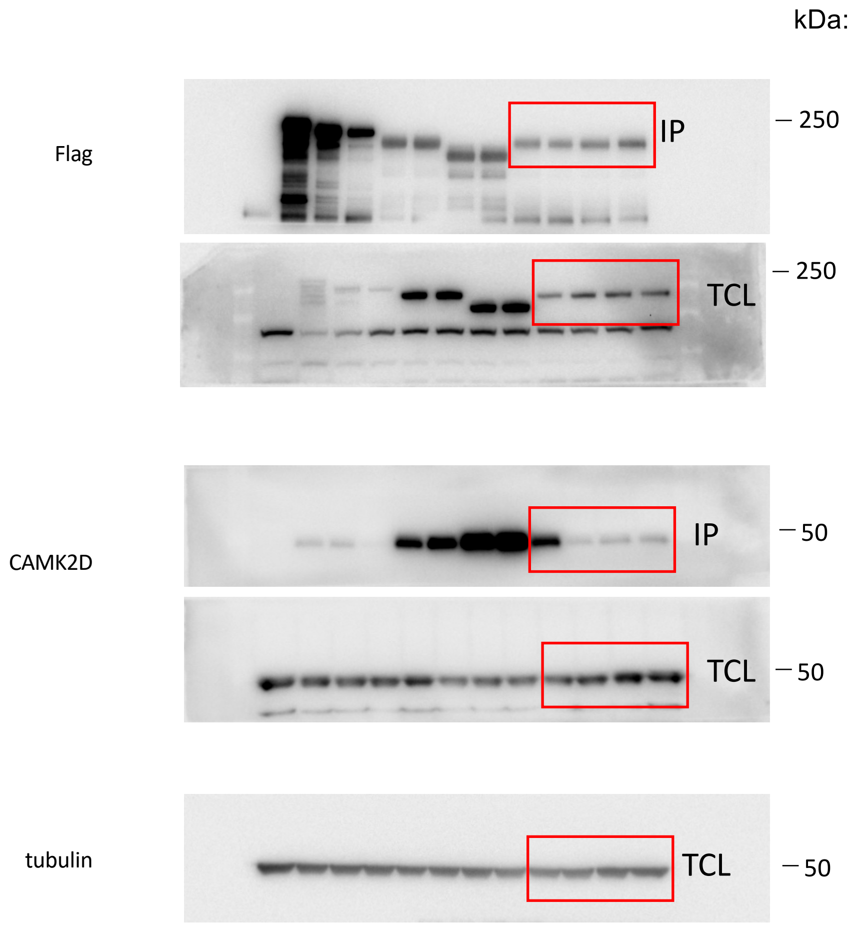

Supp Fig. 4D

n=2

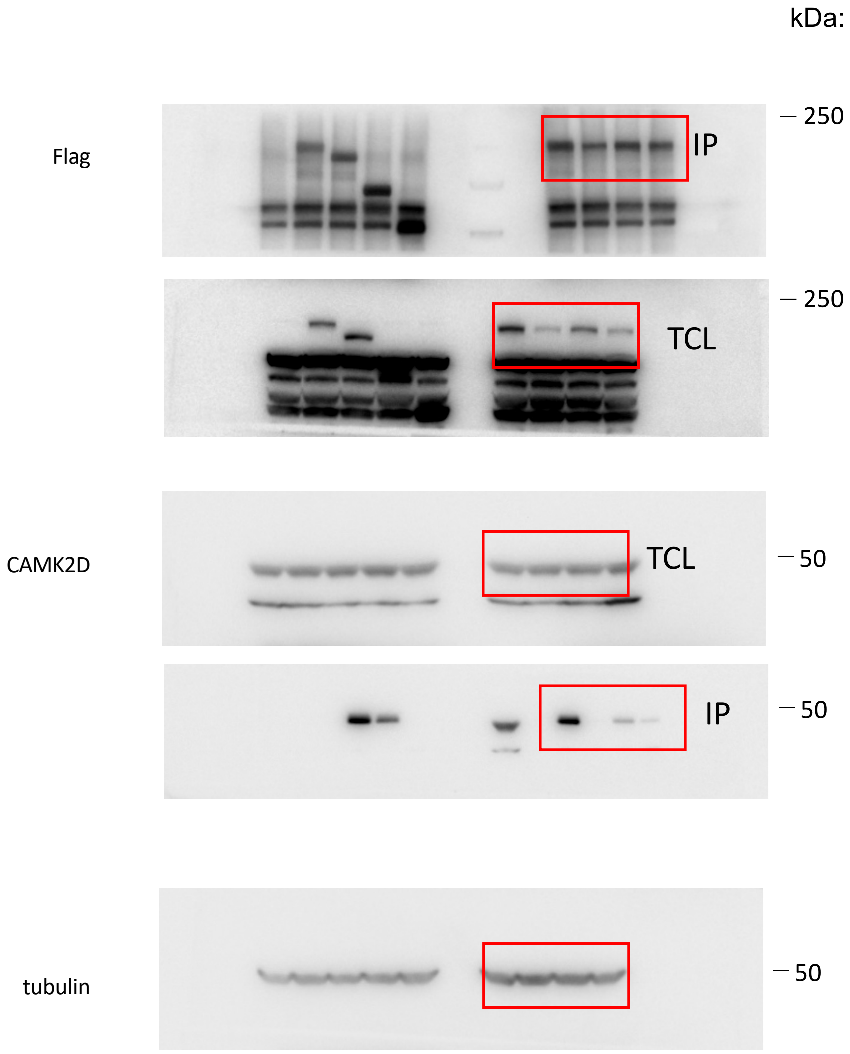

Supp Figure 4E

n=1, Shown in paper

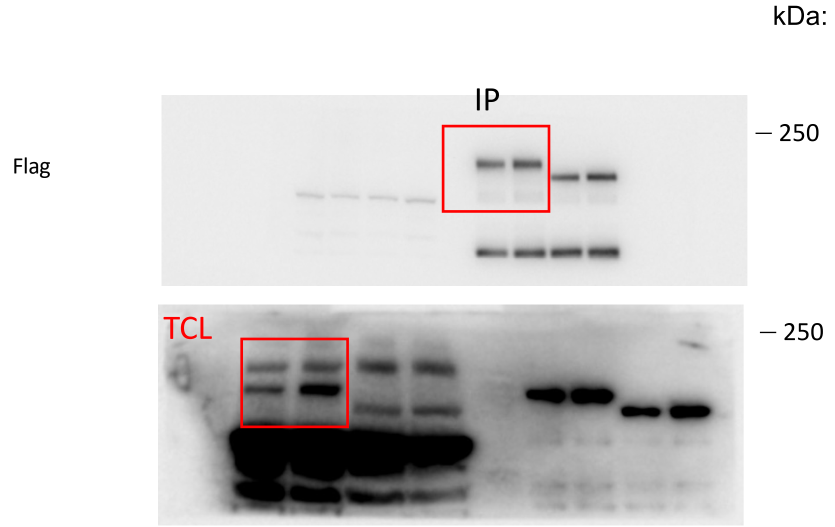

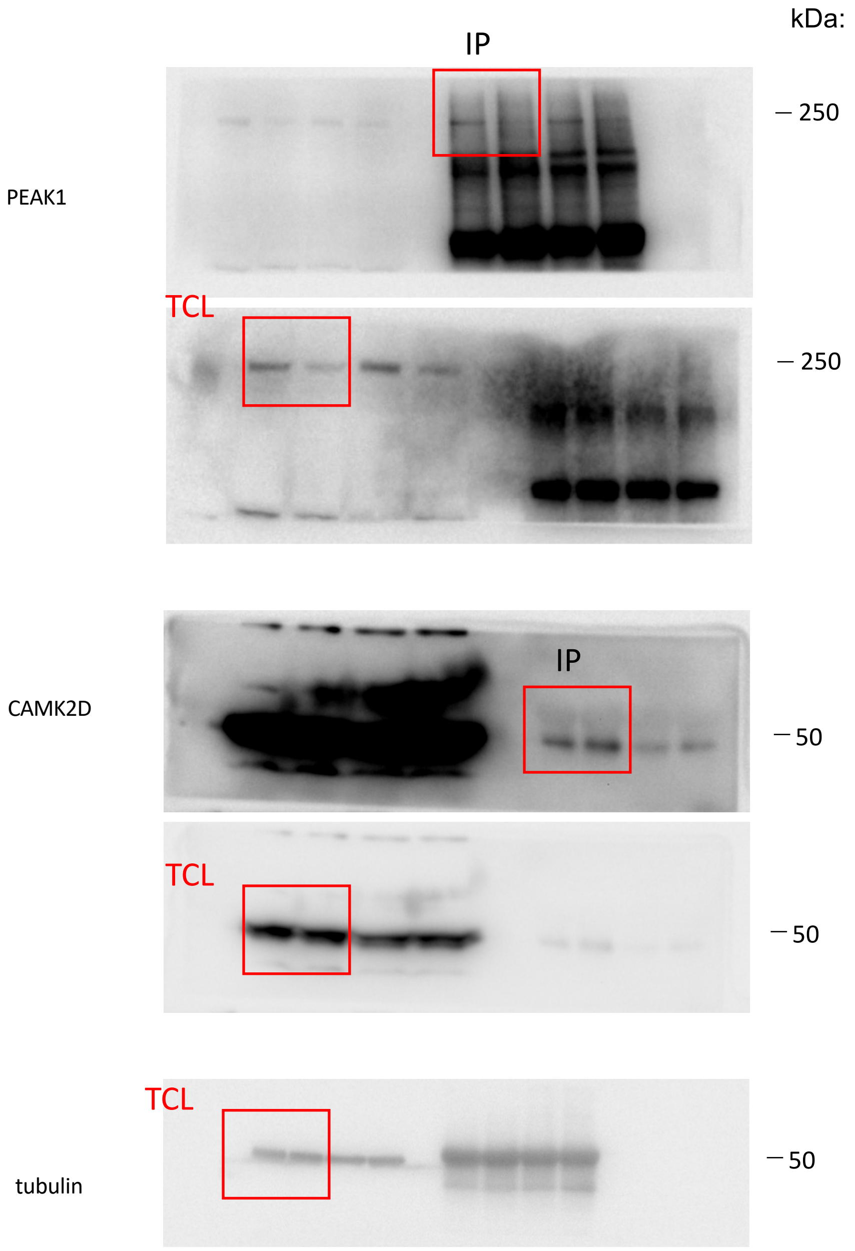

Supp Figure 4E

n=2

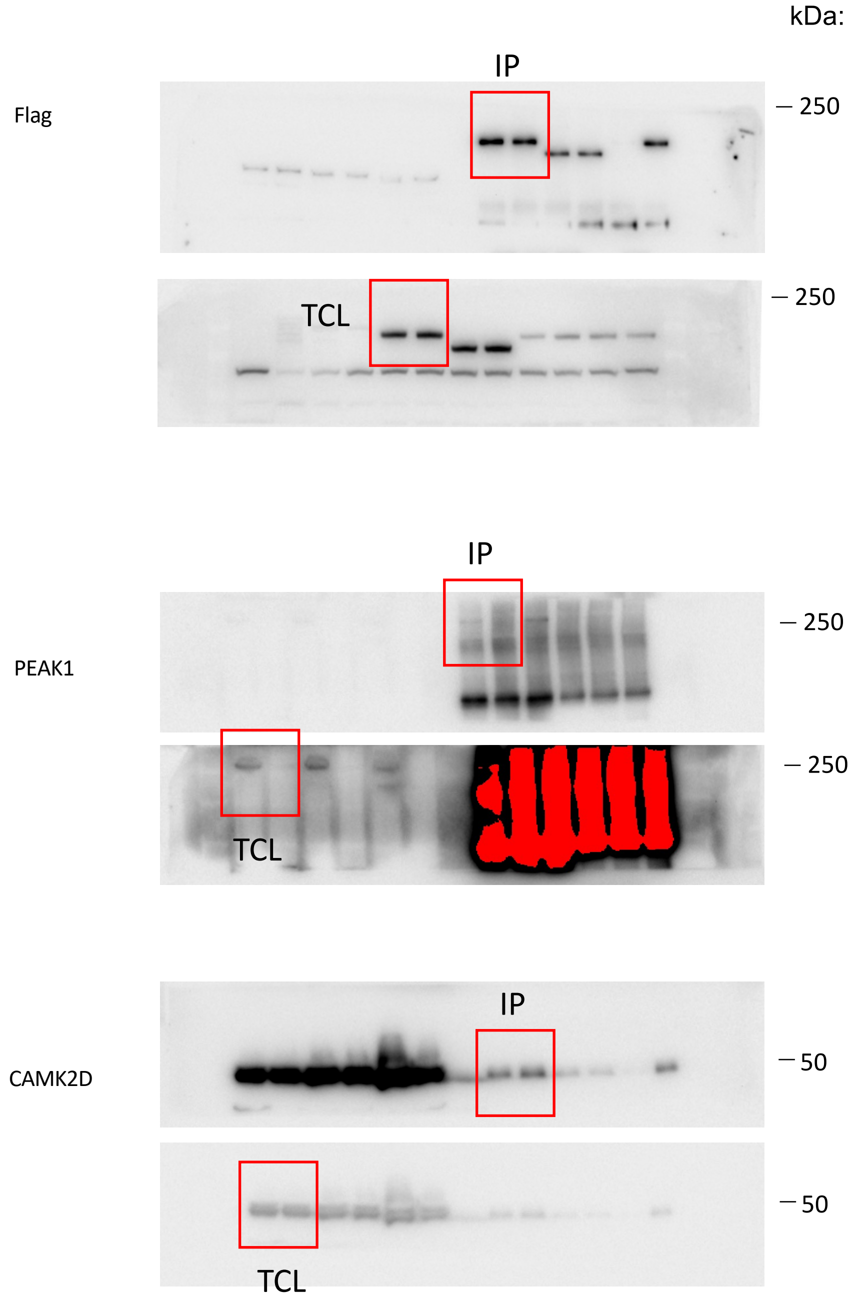

Supp Fig 7A

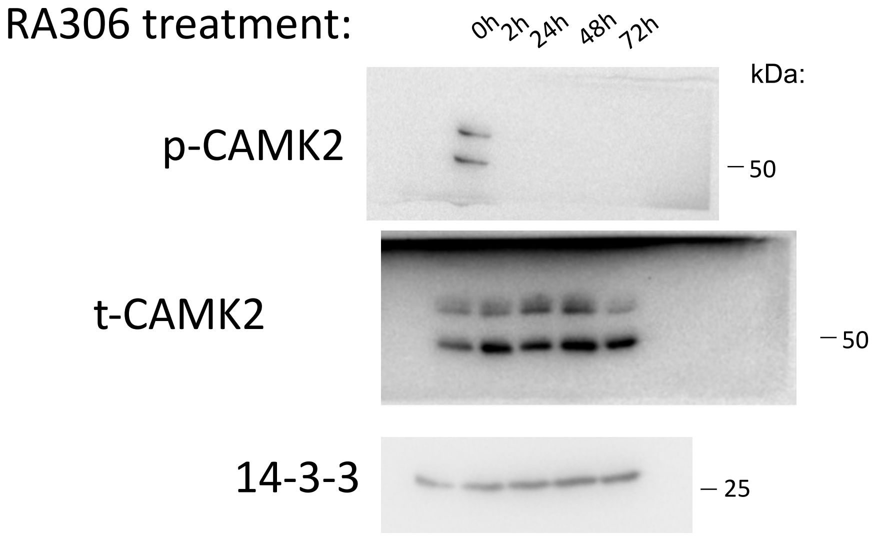

RA306 treatment:
0h
2h
24h
48h
72h
kDa:
p-CAMK2
50
t-CAMK2
50
14-3-3
25

Supp Fig 7B & C

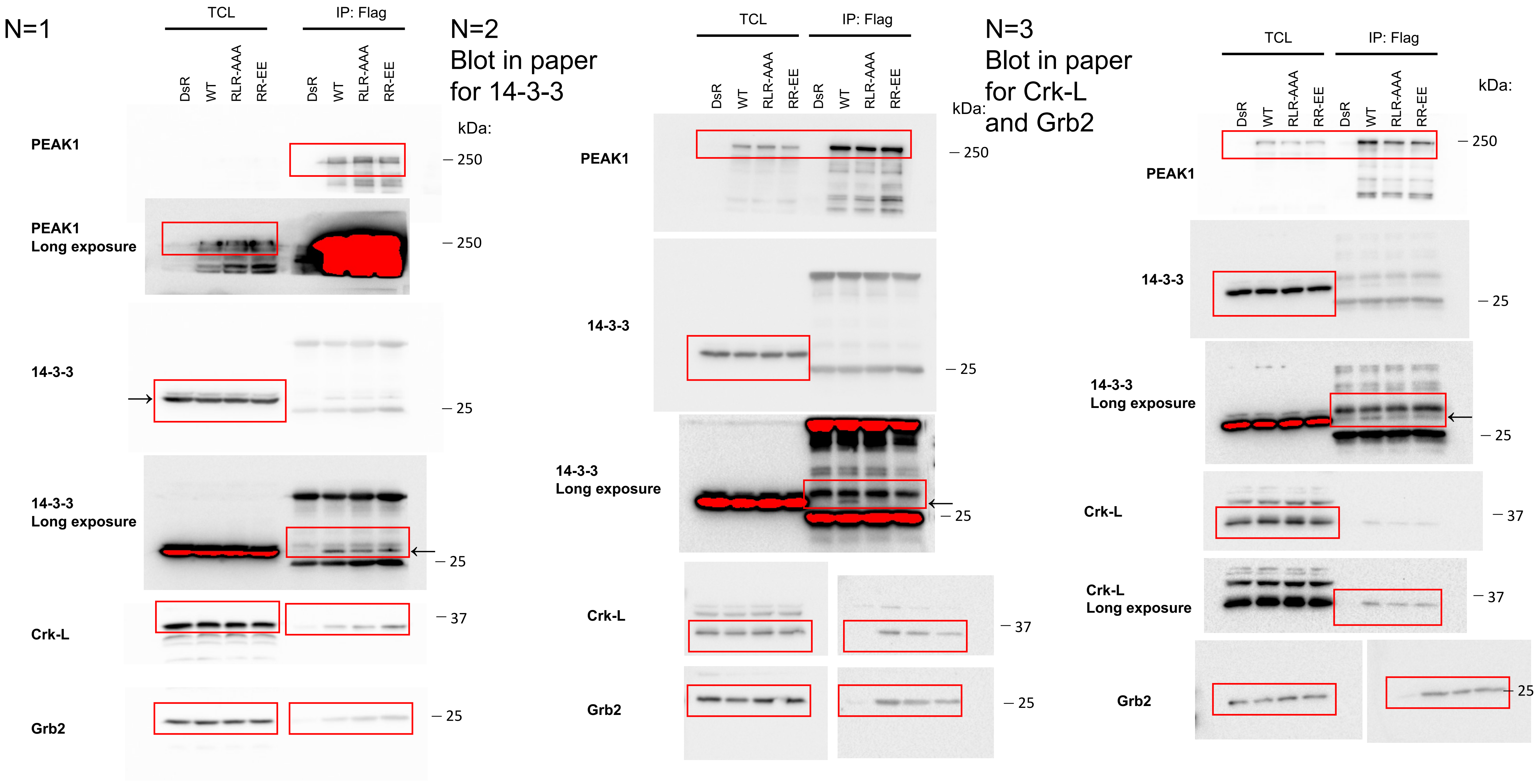

N=1
TCL
IP: Flag
DsR
WT
RLR-AAA
RR-EE
kDa:
PEAK1
250
PEAK1
Long exposure
250
14-3-3
25
14-3-3
Long exposure
25
Crk-L
37
Grb2
25
N=2
Blot in paper
for 14-3-3
TCL
IP: Flag
DsR
WT
RLR-AAA
RR-EE
kDa:
PEAK1
250
14-3-3
25
14-3-3
Long exposure
25
Crk-L
37
Grb2
25
N=3
Blot in paper
for Crk-L
and Grb2
TCL
IP: Flag
DsR
WT
RLR-AAA
RR-EE
kDa:
PEAK1
250
14-3-3
25
14-3-3
Long exposure
25
Crk-L
37
Crk-L
Long exposure
37
Grb2
25

Supp Fig 9C

N=1, used in paper

kDa:

cl.PARP

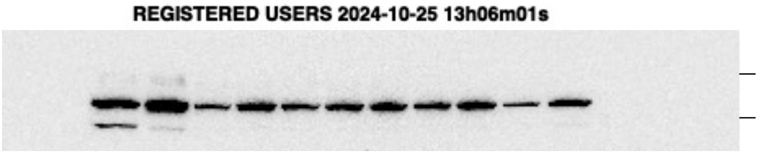

150
100

Actin

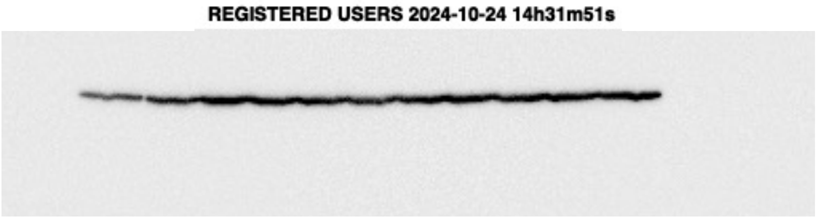

37

Supp Fig 9C

n=2

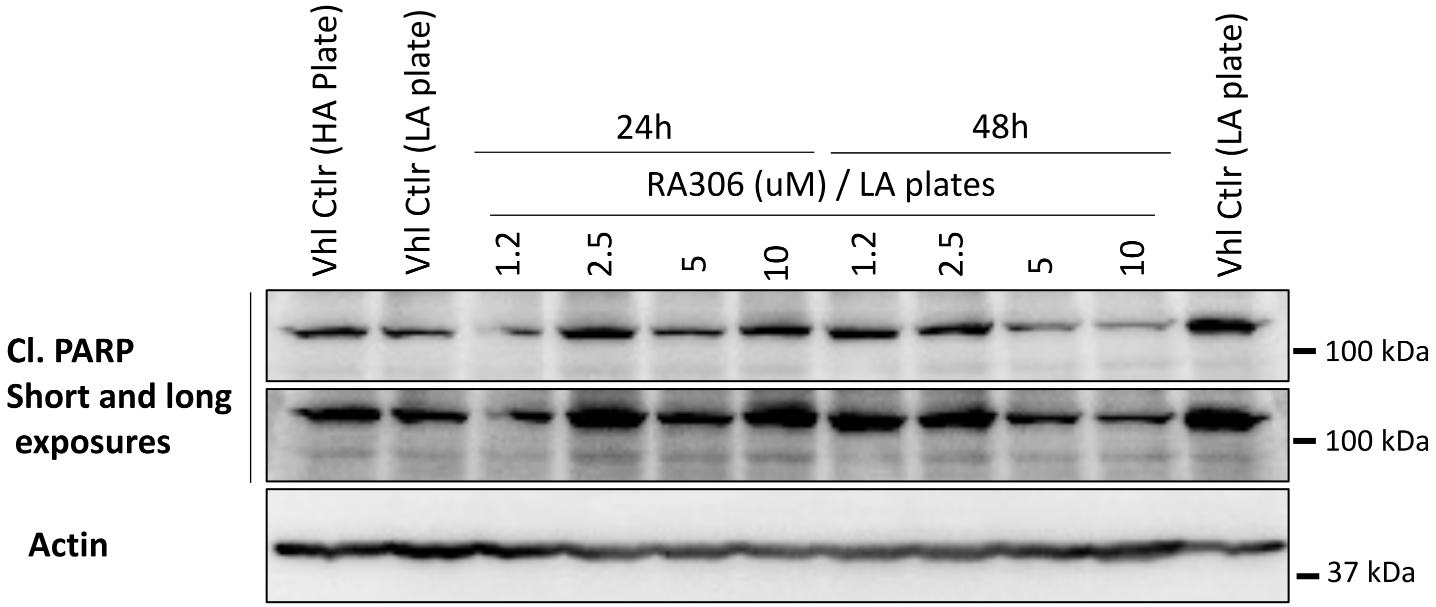

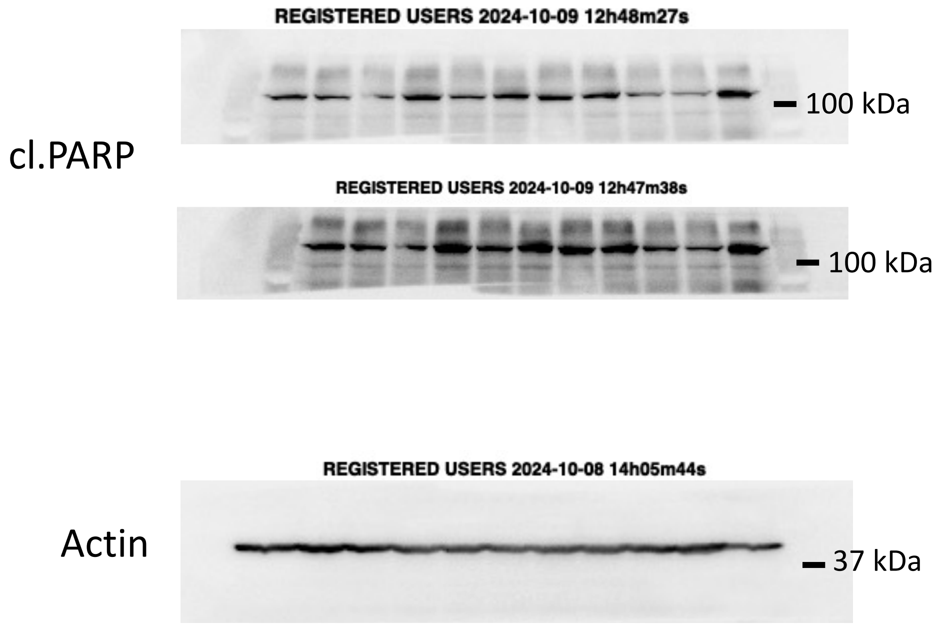

HA : High Attachment
LA : Low Attachment

Supp Fig 9D

n=1 in paper

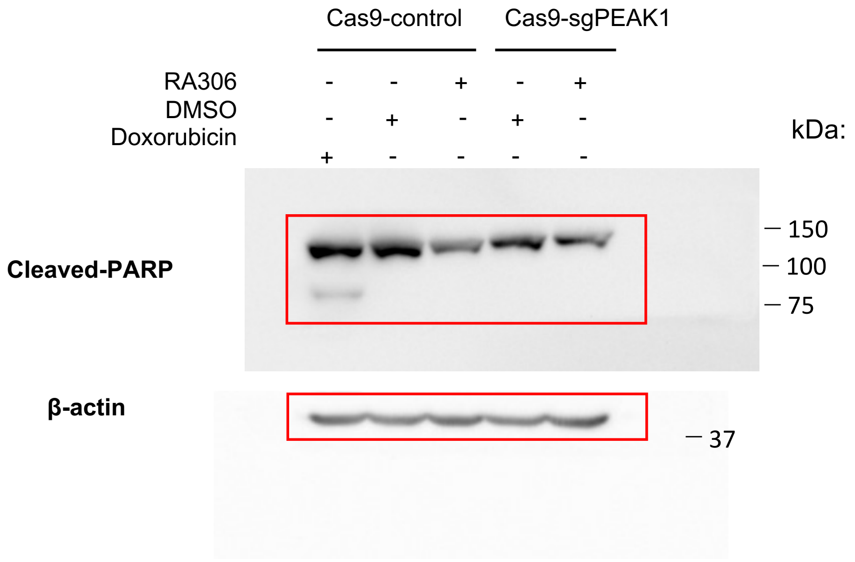

n=2

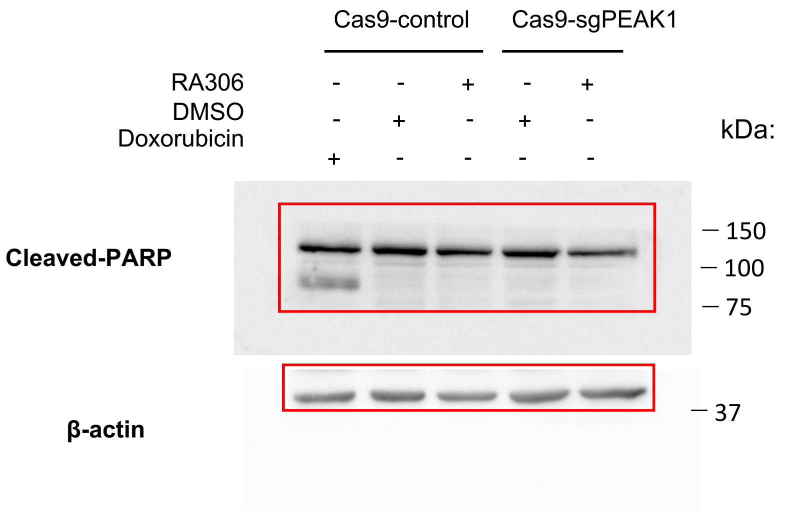

Supp Fig 10B

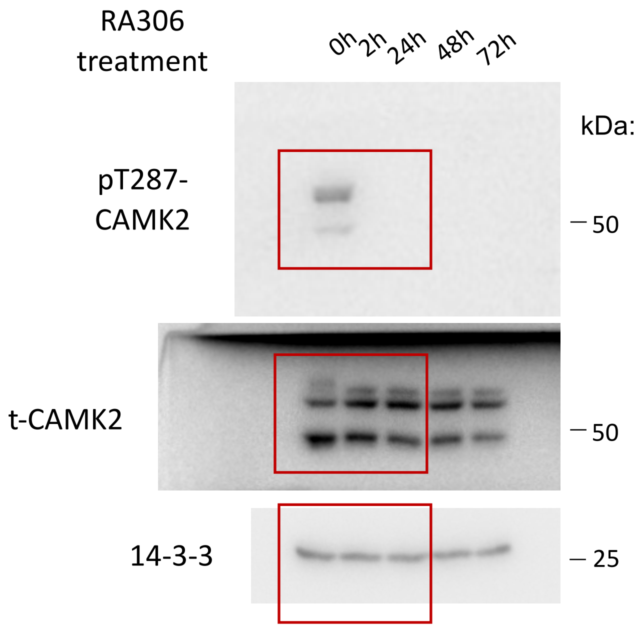
RA306
treatment
0h
2h
24h
48h
72h
kDa:
pT287-
CAMK2
50
t-CAMK2
50
14-3-3
25

Supp Fig 11A

A

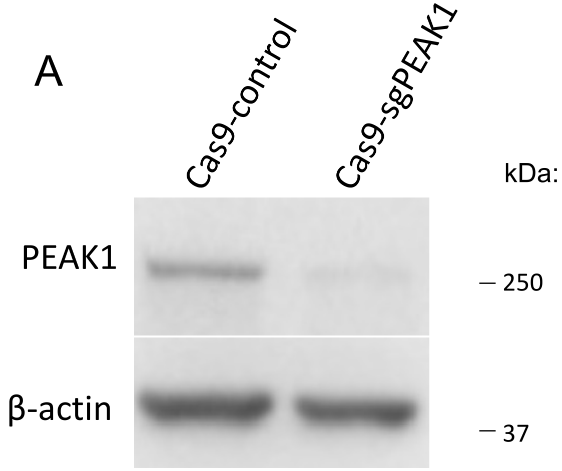

Supp Fig 11C

Boxed region indicates region of 14-3-3 panel used in figure

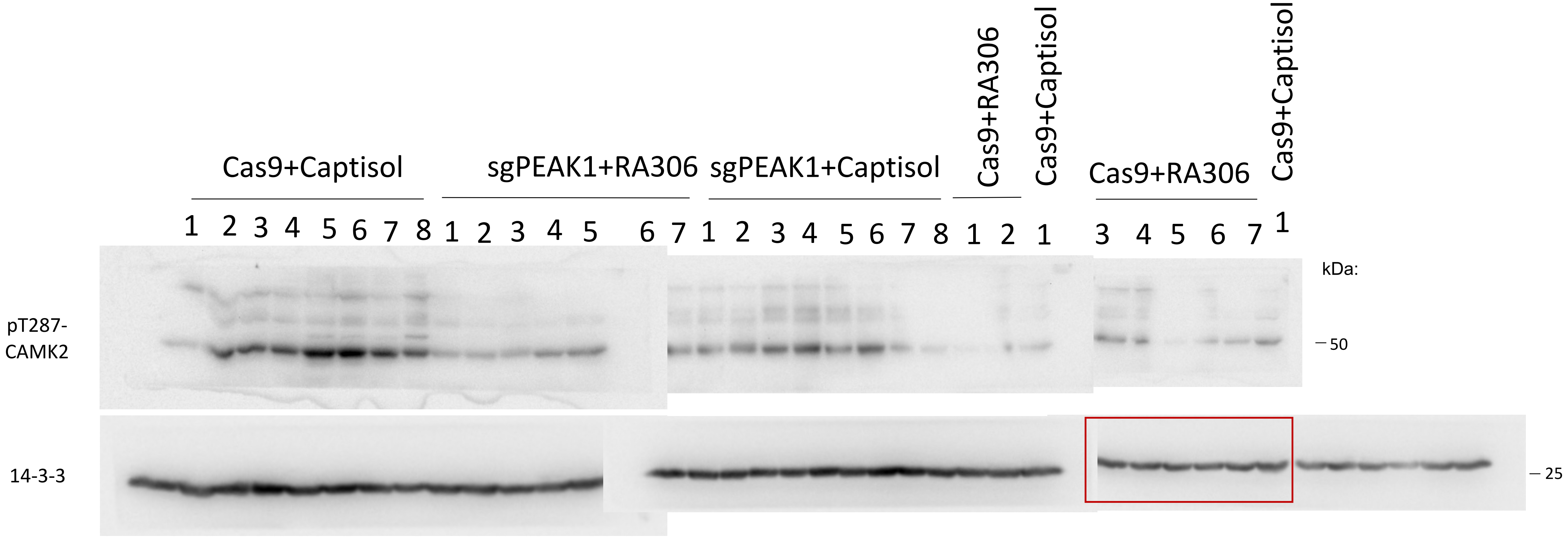

Supplement: Supplementary file 1 — Supplementary Information [file 41467_2025_57046_MOESM1_ESM.pdf]
